# Using nearly full-genome HIV sequence data improves phylogeny reconstruction in a simulated epidemic

**DOI:** 10.1038/srep39489

**Published:** 2016-12-23

**Authors:** Gonzalo Yebra, Emma B. Hodcroft, Manon L. Ragonnet-Cronin, Deenan Pillay, Andrew J. Leigh Brown, PANGEA_HIV Consortium, Christophe Fraser, Paul Kellam, Tulio de Oliveira, Ann Dennis, Anne Hoppe, Cissy Kityo, Dan Frampton, Deogratius Ssemwanga, Frank Tanser, Jagoda Keshani, Jairam Lingappa, Joshua Herbeck, Maria Wawer, Max Essex, Myron S. Cohen, Nicholas Paton, Oliver Ratmann, Pontiano Kaleebu, Richard Hayes, Sarah Fidler, Thomas Quinn, Vladimir Novitsky, ICONIC Project, Andrew Haywards, Eleni Nastouli, Steven Morris, Duncan Clark, Zisis Kozlakidis

**Affiliations:** 1 Institute of Evolutionary Biology, University of Edinburgh, Edinburgh, UK; 2 Wellcome Trust-Africa Centre for Health and Population Studies, University of KwaZulu-Natal, Durban, South Africa; 3 Department of Infectious Disease Epidemiology, Imperial College London, London, UK; 4 Wellcome Trust Sanger Institute, Hinxton, UK; 5 University of North Carolina at Chapel Hill, University of North Carolina, Chapel Hill, USA; 6 Farr Institute of Health Informatics Research, University College London, London, UK; 7 Joint Clinical Research Centre, Kampala, Uganda; 8 MRC/UVRI, Uganda Research Unit on AIDS, Entebbe, Uganda; 9 Department of Global Health, University of Washington, Seattle, WA, USA; 10 Johns Hopkins Bloomberg School of Public Health, Baltimore, MD, USA; 11 Harvard T.H. Chan School of Public Health, Boston, MA, USA; 12 MRC Clinical Trials Unit, University College London Hospital, London, UK; 13 Department of Epidemiology and Population Health, London School of Hygiene and Tropical Medicine, London, UK; 14 Department of Medicine, Imperial College London, London, UK; 15 Department of Virology, University College London Hospital, London, UK; 16 Department of Health Economics, University College London, London, UK; 17 Department of Virology, Barts Health NHS Trust, London, UK; 18 Division of Infection and Immunity, University College London, London, UK

## Abstract

HIV molecular epidemiology studies analyse viral *pol* gene sequences due to their availability, but whole genome sequencing allows to use other genes. We aimed to determine what gene(s) provide(s) the best approximation to the real phylogeny by analysing a simulated epidemic (created as part of the PANGEA_HIV project) with a known transmission tree. We sub-sampled a simulated dataset of 4662 sequences into different combinations of genes (*gag*-*pol*-*env, gag*-*pol, gag, pol, env* and partial *pol*) and sampling depths (100%, 60%, 20% and 5%), generating 100 replicates for each case. We built maximum-likelihood trees for each combination using RAxML (GTR + Γ), and compared their topologies to the corresponding true tree’s using CompareTree. The accuracy of the trees was significantly proportional to the length of the sequences used, with the *gag*-*pol*-*env* datasets showing the best performance and *gag* and partial *pol* sequences showing the worst. The lowest sampling depths (20% and 5%) greatly reduced the accuracy of tree reconstruction and showed high variability among replicates, especially when using the shortest gene datasets. In conclusion, using longer sequences derived from nearly whole genomes will improve the reliability of phylogenetic reconstruction. With low sample coverage, results can be highly variable, particularly when based on short sequences.

Most studies on HIV molecular epidemiology now use the portion of the viral *pol* gene that contains the protease (PR) and reverse transcriptase (RT) coding regions. This is because these partial *pol* sequences (around 1.3 Kb long) are routinely sequenced for genotypic resistance testing^1^,^2^,^3^. Although initially the *env* gene was considered to present the strongest phylogenetic signal, it was argued that some *env* fragments were too short and/or variable for a robust analysis^4^. After *pol* was demonstrated to accurately reconstruct HIV transmission^5^, its analysis for phylogenetic studies became the standard owing to the very large datasets available for analysis (e.g., the UK^6^ and Swiss^7^ sequence databases). In the last few years, the increasing availability of HIV whole genome sequences has made possible the analysis of other genetic regions, which has raised discussion about whether full-length genome trees should be used or which viral genes provide the best trees.

A few studies have previously approached this question by analysing HIV transmission networks in which the timing and direction of transmission were known^8^,^9^,^10^,^11^. They have suggested that the combination of more than one gene provides the best estimation of the true tree. However, all were limited to very few patients and, in some cases, short nucleotide sequences. The lack of a known, large phylogeny prevents providing a definitive comparison that would answer this question, but simulated data provide an approximation that allows having both the true tree and a recombination-free dataset.

Such data were generated in the context of the PANGEA_HIV Methods Comparison Exercise^12^ (http://www.pangea-hiv.org), for which an HIV epidemic in an African village was simulated using an agent-based model in which all sexual contacts were recorded, and those that gave rise to transmissions created a transmission tree which was recorded. Here, we used these HIV datasets to evaluate the effect of utilising viral sequence datasets of different length and from several viral genes and with different sampling depths to reconstruct the known simulated phylogenies.

## Results

**Figure 1:**
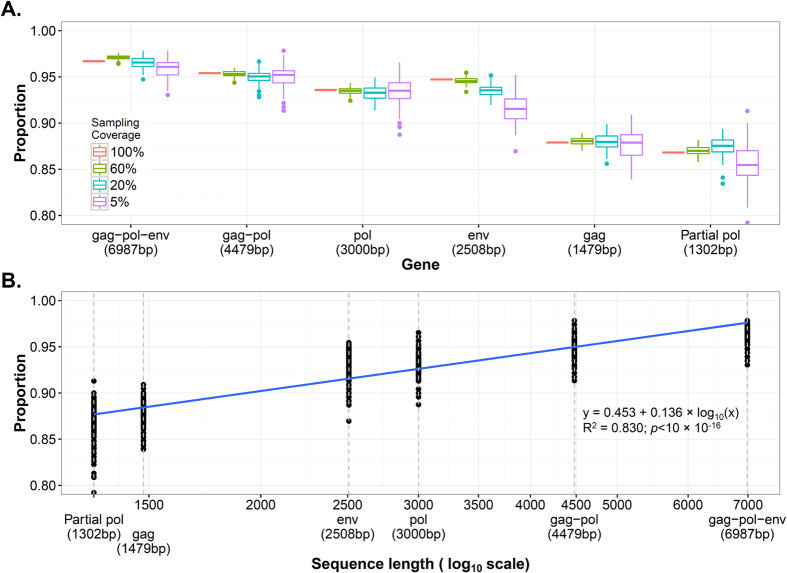
(**A**) Proportion of the maximum likelihood trees splits shared with the true tree for each gene and sampling coverage level. Genes are sorted according to length. The top and bottom limits of the boxes represent, respectively, the first and third quartiles (the distance between them represents the inter-quartile range, IQR). The lines (whiskers) include the highest and lowest values that lie within the 1.5 × IQR distance from the first and third quartiles, respectively. Data points outside this range are outliers. (**B**) Proportion of the maximum likelihood trees splits shared with the true tree according to gene length. All sampling coverage levels were considered together (see Supplementary Figure 1 for an analysis broken down by sampling coverage level). The regression line is shown in blue, for which the formula, the correlation coefficient (R^2^) and the p-value are presented. The shaded area shows the regression line’s confidence intervals. The grey, dotted vertical lines show the length of each gene considered.

From the simulated HIV sequence data generated for the PANGEA_HIV project, we produced different combinations of sampling density (100%, 60%, 20% and 5%) and viral gene use (*gag*-*pol*-*env, gag*-*pol, gag, pol, env* and partial *pol*). Sixty per cent represents approximately the sampling coverage in the UK HIV Drug Resistance Database^13^, whereas 5% represent the range in HIV sequence coverage that is believed to be relevant for cohorts in many African countries. For example, in the region of KwaZulu-Natal, South Africa, the sampling density is estimated to be between 4% and 8%, according to the specific cohort, (Prof. Tulio de Oliveira, pers. comm.). This sub-sampling was randomly replicated 100 times and ML trees were constructed, whose topology was then compared to that of the corresponding true tree. The results of the CompareTree metric (Fig. 1A) show that the proportion of correct tree splits increased with the length of the sequences used. The genome datasets showed the best performance considering all the sampling coverage levels together (Table 1), with an average metric value of 0.965 (95% confidence interval (CI) = 0.964–0.966). It was closely followed by *gag*-*pol* (0.951 [0.950–0.952]), *pol* (0.934 [0.933–0.935]) and *env* (0.932 [0.930–0.933]) in that order. The smaller *gag* (0.879 [0.877–0.880]) and partial *pol* (0.867 [0.866–0.869]) sequences showed the worst performances.

Thus, the proportion of correct tree splits increased in direct proportion to the length of the sequences used. A linear regression analysis showed a statistically significant positive correlation between the metric and a logarithmic transformation of the sequence length, yielding a correlation value of R^2^ = 0.83 (p < 10^−16^; see also Fig. 1B for the complete formula). This was also true when analysing the sampling coverage levels individually (R^2^ > 0.78 and p < 0.01 for all levels; see also Supplementary Figure 1). However, when considering specific genes, the analysis of the *env* gene (length = 2508 bp) was more accurate than that of *pol* (length = 3000 bp) when reconstructing the true tree in the 100% (point estimation=0.947 versus 0.936), 60% (mean or the replicates = 0.946 [95%CI = 0.945–0.945] versus 0.935 [0.934–0.935]; Student’s t-test p < 10^−16^) and 20% (mean of the replicates = 0.935 [95%CI = 0.934–0.936] versus 0.933 [0.931–0.934]; p = 0.01) sampling levels, but it showed more variability and worse results than the *pol* analyses in the replicates with 5% sampling level: mean = 0.915 (95%CI = 0.912–0.918) in *env* versus mean = 0.936 (95%CI = 0.933–0.938) in *pol* (p < 10^−16^). In general, *env* was the gene that showed the largest difference in the mean estimations across the different sampling coverage levels.

In the subsampled datasets, the 60% sampling coverage dataset performed very similarly to the fully sampled dataset, even showing means significantly higher than the 100% sampling coverage estimates when analysing the *gag*-*pol*-*env* (0.971 [95%CI = 0.970–0.971] versus 0.967; p < 10^−16^), *gag* (0.880 [0.879–0.881] versus 0.879; p = 6.5 × 10^−3^) and partial *pol* datasets (0.870 [0.869–0.871] versus 0.868; p = 1.6 × 10^−4^).

In the 20% sampling level there was considerable overlap in performance among the larger fragments, but that of the smaller regions was substantially poorer. With 5% sampling coverage levels, the results showed the largest confidence intervals, revealing a substantial variability among the replicates, although some of these replicates outperformed estimations from the levels with higher sampling coverage.

Although quantitatively small, these differences in accuracy of tree reconstruction are important for identifying transmission clusters. We tested the impact of these differences using a standard methodology to detect transmission networks from the trees generated in this study by comparing the proportion of clusters found in the true tree (“true clusters”) that were also found when analysing the ML trees. We did this using the *gag-pol-env* sequence and the partial *pol* sequences (as is the norm in the vast majority of studies) in the 100% sampled dataset, and we discovered that the use of *gag-pol-env* detected a significantly higher proportion of true clusters (778 out of 788 true clusters in *gag-pol-env* (98.73%) versus 774 out of 827 true clusters in partial *pol* (93.59%), chi-square test p = 1.95 × 10^−7^). Thus, even in the fully sampled dataset, the reconstruction of trees from partial sequences implies a significant and important difference in the outcome.

## Discussion

We have used simulated HIV sequence data to show how the use of genes of different lengths can affect the correct reconstruction of the true viral phylogeny. The proportion of correct trees increased in almost direct proportion to the length of the sequences used. Thus, the 7 Kb *gag*-*pol*-*env* nearly full-genome sequences were best at reconstructing the true tree.

The 60% sampling coverage provides the most similar results to the analyses of the complete datasets, which emphasises the superior reliability of studies based on high densely sampled epidemics. In contrast, lower sampling depths (20% and 5%, which resemble the sampling settings found in Africa and developing areas) greatly reduced the accuracy of tree reconstruction –visible in the high variability between the replicates– especially when using the short clinical *pol* dataset.

We presumably obtained values higher than expected in a real-world analysis, particularly because there is a complete fit between the evolutionary model used to simulate the sequence data and the model used for analysing it. In addition, the good performance of the *env* analyses is partly due to the fact that its characteristic insertion/deletion variation was not simulated. Nevertheless the fact that *env* trees can outperform the *pol* trees, suggests that, in principle, the higher evolutionary rate in *env* can improve reconstruction.

Here we used a metric that is proportional to the RF metric –the most widely used method to estimate the distance/similarity between two phylogenetic trees. While this might be a simplistic metric, it is an intuitive and powerful method to compare trees, although its limitation is that it does not provide a means to state that one tree is significantly more similar to the true tree than a second tree is.

Our results demonstrate that the length of the sequence increases the reliability of phylogeny reconstruction in simulated data. In the simulations, different evolutionary rates applied to the *gag-pol* and *env* genes, as seen in real datasets. These were of 1.91 × 10^−3^ for *gag-pol* (or *pol*) and 3.83 × 10^−3^ for *env*, i.e. the evolutionary rate for *env* was twice that of *gag-pol*. Thus, the amount of variation that we find in *env* (length = 2508 nt) would be equivalent to an approximately 5 Kb-long *gag-pol* sequence. This could explain that, in some replicates, *env* outperforms *pol* (length = 3000 nt). However, there was no insertion/deletion variation in the simulated sequences and in analysing real datasets this apparent superiority of *env* over more conserved genes is constrained by errors in alignment if hypervariable regions are included.

Although we did not perform a bootstrapping analysis of the reconstructed trees, previous analyses have further demonstrated that support for groupings in the tree is increased when longer sequences are used, and clustering found in full-length datasets can be missed when using sub-genomic regions^14^,^15^,^16^. Given the difficulty in generating and/or handling full genome datasets, our results demonstrate that *gag*-*pol* provides a dependable approximation; however it should be kept in mind that, at this point and considering we analysed a simulated dataset, the good performance of *gag*-*pol* could be more attributable to these genes’ combined length than to their particular characteristics.

In conclusion, thanks to the more affordable generation of full HIV genomes, as is the goal of the PANGEA_HIV consortium^17^, the use of longer genetic regions (such as concatenated *gag, pol* and *env* or *gag*-*pol*) will allow for a more reliable reconstruction of transmission events. The traditional short *pol* sequences generated for resistance testing that are used in most molecular epidemiology studies are substantially less reliable, especially with low sampling depths. An effort to generate highly sampled datasets is also needed to increase our ability to reconstruct real HIV epidemics.

## Methods

### HIV epidemic simulation

The PANGEA_HIV phylodynamic Methods Comparison Exercise^12^ (http://www.pangea-hiv.org/Projects#phylodynamic) created a simulation resembling an African Village, which was based on high- and low-risk households and a small sex worker group. These simulations made use of the Discrete Spatial Phylo Simulator adapted to HIV-specific components (DSPS-HIV), which is an individual-based stochastic simulator. Using a specifiable contact network, the DSPS-HIV models HIV transmissions and records all sexual contacts. Selecting those which gave rise to transmissions produced the transmission tree. To generate the HIV sequences associated to these transmissions events, viral phylogenies that reflect between- and within-host viral evolution were simulated down the transmission tree using VirusTreeSimulator (https://github.com/PangeaHIV/VirusTreeSimulator).

In order to reconstruct ancestral subtype C sequences to be used as starting point of the simulation, a dataset of Southern African full genome subtype C sequences was downloaded from Los Alamos database (http://www.hiv.lanl.gov/). It included 100 sequences selected to represent a balanced number of sequences per calendar year (1989–2011), and were sampled in South Africa (n = 46), Botswana (n = 41), Zambia (n = 8) and Malawi (n = 5). The GenBank accession numbers corresponding for these 100 sequences are provided in the Supplementary Table 1. This dataset was separated into *gag, pol* and *env* and ancestral sequences for each gene were reconstructed using BEAST v1.8.1^18^ applying GTR + 4Γ + I as nucleotide substitution model and Bayesian skyride as demographic model.

These ancestral sequences were used as starting point to simulate sequences along these viral phylogenies using πBUSS^19^, with substitution rates parameterized from the aforementioned analyses of Southern African sequences. To increase realism, different substitution rates applied to different genes (with a rate twice as high for *env* as for *gag* and *pol*) and different codon positions (1st and 2nd vs 3rd). Finally, the simulations were parameterized to emulate prevalence and incidence estimates from the peak of the African HIV epidemic in the late 1980s-early 1990s^20^,^21^,^22^, before treatment roll-out, so the date of the root of the sequences coincides with the subtype C common ancestor in the 1940s^23^.

More specific information about the sequence simulation is provided in the following PANGEA_HIV document: https://www.dropbox.com/sh/zlv40u4vnmpvy71/AAC8-yTPJA74OcYzvTCTb-H2a/201502/Village_unblinded/DSPS-Feb15Release-Details.pdf?dl=0.

### Analysis dataset

We sampled all HIV simulated sequences corresponding to all infected individuals (one sequence per individual) in a 5-year period –between years 40 and 45 after the simulated epidemic started. From these simulated HIV sequences we created different combinations of sequence sampling depths and genomic regions. The full dataset contained 4662 sequences, and we adopted sub-sampling levels of 60% 20% and 5% sampling density which therefore included, respectively, 2798, 933 and 233 sequences. These sequences were chosen at random from the dataset with 100% sampling coverage. For the 60%, 20% and 5% sampling coverage levels we generated 100 independent sub-samples to test the reproducibility of the analyses.

We split each of these sequence datasets into: (1) “genome” (which represented the concatenation of *gag, pol* and *env* (6987 bp)), (2) *gag*-*pol* (4479 bp), (3) *gag* (1479 bp), (4) complete *pol* (3000 bp), (5) *env* (2508 bp), and (6) partial *pol* (1302 bp, the region commonly generated for PR + RT resistance testing).

The fully-sampled simulated sequence dataset as well as the true transmission tree are available at http://hiv.bio.ed.ac.uk/datasets/Yebra2016_Tree_Comparison_dataset.zip.

### Phylogenetic tree comparison

We obtained the top-scoring maximum likelihood (ML) tree for each of these datasets using RAxML v8.2^24^ under the GTR + Γ substitution model. For the nearly full genome trees, we applied a partition analysis in RAxML to accommodate for different evolutionary models in *gag*-*pol* versus *env*.

The Robinson-Foulds (RF)^25^ metric is the most widely used measure of phylogenetic tree similarity. Given two phylogenetic trees, this metric counts the number of splits or clades induced by one of the trees but not the other. Here, we use an approximation to the RF metric implemented in the CompareTree program (http://meta.microbesonline.org/fasttree/treecmp.html), which also calculates the fraction of splits in the query tree (i.e., the reconstructed trees) that are shared with the reference one (i.e., the true trees). Unlike the RF metric, this value represents a proportion (therefore it ranges from 0 to 1), providing a metric that is more intuitive and easier to interpret and compare. We use the proportion of shared splits as an indicator of the fidelity in reconstructing the corresponding, sub-sampled true tree.

Finally, in order to evaluate the implications of the topology differences, a phylogenetic cluster comparison analysis was performed in the fully sampled dataset using the Cluster Picker and Cluster Matcher programs^26^.

### Statistical analyses

We compared the results from different genes and/or sampling coverage levels by using a two-sample Student’s t-test. When comparing to the fully sampled datasets (100% sampling coverage), for which only point estimations were obtained because replicates cannot be produced, a one-sample t-test was performed to test whether the corresponding mean distribution was significantly different than the point estimation of the 100% sampling coverage level. Finally, we applied a linear regression analysis to explore the relationship between the results and the sequence length. All this calculations were produced in R^27^ version 3.1.2.

## Additional Information

**How to cite this article**: Yebra, G. *et al*. Using nearly full-genome HIV sequence data improves phylogeny reconstruction in a simulated epidemic. *Sci. Rep.* **6**, 39489; doi: 10.1038/srep39489 (2016).

**Publisher's note:** Springer Nature remains neutral with regard to jurisdictional claims in published maps and institutional affiliations.

## Supplementary Material

Supplementary Information

## Figures and Tables

**Table 1:** Proportion of the maximum likelihood trees splits shared with the true tree according to gene and sampling coverage level.

Gene	Length (bp)	Sampling coverage level (mean [95% confidence interval])				
		All	100%	60%	20%	5%
*gag-pol-env*	6987	0.965 (0.964–0.966)	0.967	0.971 (0.970–0.971)	0.965 (0.964–0.966)	0.959 (0.957–0.961)
*gag-pol*	4479	0.951 (0.950–0.952)	0.954	0.953 (0.953–0.954)	0.950 (0.948–0.951)	0.950 (0.948–0.953)
*pol*	3000	0.934 (0.933–0.935)	0.936	0.935 (0.934–0.935)	0.933 (0.931–0.934)	0.936 (0.933–0.938)
*env*	2508	0.932 (0.930–0.934)	0.947	0.946 (0.945–0.946)	0.935 (0.934–0.936)	0.915 (0.912–0.918)
*gag*	1479	0.879 (0.877–0.880)	0.879	0.880 (0.879–0.881)	0.880 (0.878–0.881)	0.877 (0.873–0.880)
Partial *pol*	1302	0.867 (0.866–0.869)	0.868	0.870 (0.869–0.871)	0.875 (0.873–0.877)	0.857 (0.853–0.861)

The table shows the mean value and its 95% confidence intervals for the 100 replicates performed in each case. Note that for the full dataset (100% sampling coverage) only one estimation is shown because no replicates can be performed. The genes are ordered in descending order of sequence length.
