# Supplementary material for: Using nearly full-genome HIV sequence data improves phylogeny reconstruction in a simulated epidemic
Source: Sci Rep. 2016 Dec 23;6:39489. doi: 10.1038/srep39489 (PMC5180198; doi:10.1038/srep39489)
Supplement: Supplementary Information [file srep39489-s1.pdf]

Supplementary information

Using nearly full-genome HIV sequence data improves phylogeny reconstruction in a simulated epidemic

Gonzalo Yebra[1,*], Emma B. Hodcroft[1], Manon Ragonnet-Cronin[1], Deenan Pillay[2] & Andrew J. Leigh Brown[1] on behalf of the PANGEA_HIV Consortium & the ICONIC Project.

[1] Institute of Evolutionary Biology, University of Edinburgh, Edinburgh, UK

[2] Wellcome Trust-Africa Centre for Health and Population Studies, University of KwaZulu-Natal, Durban, South Africa

* Corresponding author: Gonzalo.Yebra@ed.ac.uk (GY)

This file contains:

- Supplementary Figure 1
- Supplementary Table 1

Supplementary Figure 1. Proportion of the maximum likelihood trees splits shared with the true tree according to gene length and sampling coverage level.

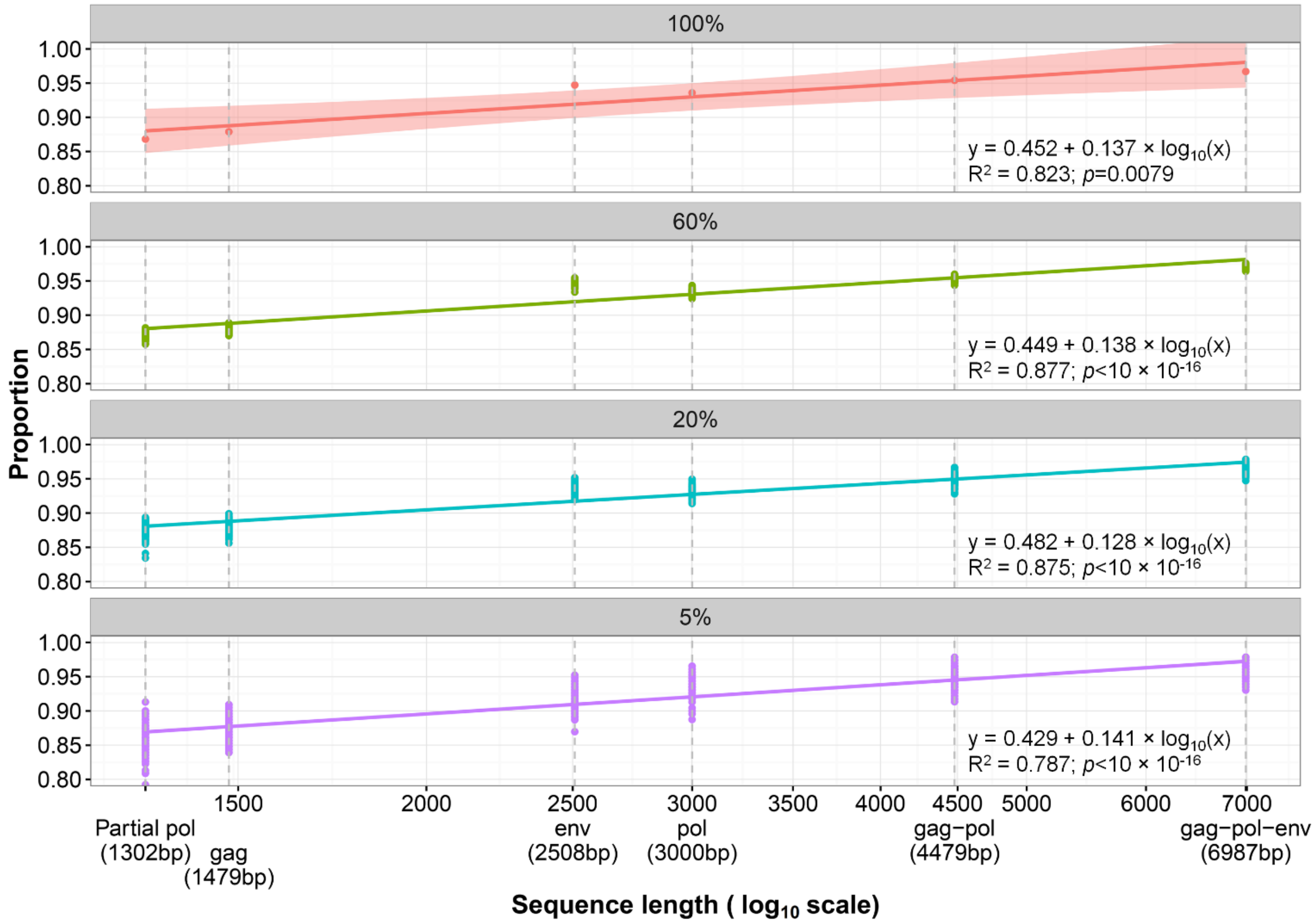

The regression lines are shown, for which the formula, the correlation coefficient (R^2) and the p-value are presented for each sampling coverage level. The shaded area shows the regression lines' confidence intervals (note the wider confidence for the 100% sampled dataset owing to the fact that it only includes one estimation per gene). The grey, dotted vertical lines show the length of each gene considered.

Supplementary Table 1. Southern African HIV-1 subtype C full-length sequences selected from Los Alamos National Laboratory HIV Database and used to reconstruct ancestral subtype C sequences.

	Accession number	Sampling country	Sampling year
1	AF443074	Botswana	1996
2	AF443075	Botswana	1996
3	AF443076	Botswana	1998
4	AF443077	Botswana	1998
5	AF443078	Botswana	1998
6	AF443079	Botswana	1998
7	AF443080	Botswana	1998
8	AF443081	Botswana	1998
9	AF443082	Botswana	1998
10	AF443083	Botswana	1999
11	AF443084	Botswana	1999
12	AF443085	Botswana	1999
13	AF443086	Botswana	1999
14	AF443087	Botswana	1999
15	AF443088	Botswana	2000
16	AF443089	Botswana	2000
17	AF443090	Botswana	2000
18	AF443091	Botswana	2000
19	AF443092	Botswana	2000
20	AF443093	Botswana	2000
21	AF443094	Botswana	2000
22	AF443095	Botswana	2000
23	AF443096	Botswana	2000
24	AF443097	Botswana	2000
25	AF443098	Botswana	2000
26	AF443099	Botswana	2000
27	AF443100	Botswana	2000
28	AF443101	Botswana	2000
29	AF443102	Botswana	2000
30	AF443103	Botswana	2000
31	AF443104	Botswana	2000
32	AF443105	Botswana	2000
33	AF443107	Botswana	2000
34	AF443108	Botswana	2000
35	AF443109	Botswana	2000

Supplementary Table 1 (continued)

	Accession number	Sampling country	Sampling year
36	AF443110	Botswana	2000
37	AF443111	Botswana	2000
38	AF443112	Botswana	2000
39	AF443113	Botswana	2000
40	AF443114	Botswana	2000
41	AF443115	Botswana	2000
42	KC156119	Malawi	2007
43	KC156114	Malawi	2007
44	KC156216	Malawi	2008
45	KF527172	Malawi	2008
46	KC156214	Malawi	2009
47	JN188292	South Africa	1990
48	AY118165	South Africa	1997
49	BD437615	South Africa	1998
50	EU293446	South Africa	1999
51	AY228556	South Africa	1999
52	AY585268	South Africa	2000
53	AY463217	South Africa	2000
54	AY228557	South Africa	2001
55	DQ369995	South Africa	2002
56	DQ396380	South Africa	2003
57	AY772700	South Africa	2003
58	AY901969	South Africa	2003
59	AY901975	South Africa	2003
60	DQ056411	South Africa	2003
61	DQ164113	South Africa	2003
62	DQ093601	South Africa	2003
63	DQ093593	South Africa	2003
64	DQ093596	South Africa	2003
65	DQ093589	South Africa	2003
66	DQ056408	South Africa	2003
67	AY901967	South Africa	2003
68	AY878061	South Africa	2003
69	GQ999975	South Africa	2004
70	AY703909	South Africa	2004
71	AY878058	South Africa	2004
72	AY901976	South Africa	2004

Supplementary Table 1 (continued)

	Accession number	Sampling country	Sampling year
73	DQ093595	South Africa	2004
74	DQ164126	South Africa	2004
75	DQ445631	South Africa	2004
76	DQ396387	South Africa	2004
77	DQ011170	South Africa	2004
78	DQ011180	South Africa	2004
79	DQ369992	South Africa	2005
80	DQ369982	South Africa	2005
81	DQ396372	South Africa	2005
82	GQ999991	South Africa	2005
83	GQ999987	South Africa	2005
84	GQ999983	South Africa	2005
85	GQ999976	South Africa	2005
86	KC156130	South Africa	2007
87	KC156127	South Africa	2007
88	KC156125	South Africa	2007
89	JX140666	South Africa	2008
90	KC156221	South Africa	2008
91	JX140667	South Africa	2009
92	JX140669	South Africa	2010
93	AB485647	Zambia	1989
94	AF286225	Zambia	1996
95	AB254153	Zambia	2002
96	AB254149	Zambia	2002
97	AB254148	Zambia	2002
98	FJ496214	Zambia	2003
99	KF716466	Zambia	2009
100	KF716467	Zambia	2011
